# Diphtherite, or Membranous Croup, Treated by Ammonia and Oil

**Published:** 1849-07

**Authors:** T. L. Ogier


### ARTICLE II.

### *Diphthritee or Membranous Sore Throat, treated by Ammonia and Oil.* By T, L. OGIER, M.D.

Membranous sore throat being one of the most fatal diseases to which childrenare liable, it will not be uninteresting to give a slight history of the following case, treated successfully by volatile liniment given internally.

On the 12th December I was called to see a little girl, three years of age, the daughter of Mr. T., said to be affected with croup. The little patient was breathing with great difficulty with occasional cough, attended with the regular barking sound characteristic of croup, the pulse was very frequent and the skin hot. Upon examination of the throat, the fauces and palate were seen covered with the whitish membrane, establishing perfectly the nature of the disease. The account given by the mother was, that for three days past the child seemed to have a very bad cold and the hoarseness had gradually come on to its present state. Squills had been given occasionally, but, as the hoarseness increased, hive syrup and hippo tea were administered, until free vomiting was produced. These remedies failed to produce any relief, and upon my visit, I found the patient as above described.

In conversing with my friend Dr. Lebby, some time since concerning this disease and the inefficiency of all modes of treatment in arresting its progress, he related several cases of very marked success attending the treatment by ammonia and oil—volatile liniment—recommended to him by Dr. J. A. Johnson. Having seen no very decided benefit from any one remedy hitherto used, I thought this a favorable case in which to use the ammonia. I directed the throat to be rubbed externally with aq. ammonia, and a flannel wet with the same to be kept around the neck, and the following mixture given: ℞ aq. ammoniæ, ol. olivæ, mellis, equal parts. A tea-spoonful to be given every two or three hours. This mixture was given with some difficulty, the child seeming to experice pain when the mixture was swallowed—it produced free vomiting. This remedy was commenced about 12 o'clock in the day, and continued every two or three hours until 9 o'clock, P. M. A dose of calomel, grs. vi, was then given. This operated during the night three or four times.

The operations were perfectly green and without smell. At my visit early the next morning, Dec. 14th, the hoarseness was the same and the breathing difficult, the pulse continued frequent and the skin hot, but slightly moist. The hartshorn applied externally to the neck had blistered it, I therefore discontinued the application and directed a bread poultice to be continued as before. In the afternoon I found my patient had vomited frequently during the day, and in the matter vomited were seen several portions of the false membrane; the breathing was better and the hoarseness less; the fauces and velum were still white and covered with membrane. Two grains of calomel combined with a half grain of ipecac. were ordered at night. This operated once—the next morning—and the stool presented the same green appearance as before, but had somewhat of a fœcal smell. The mixture to be continued in doses as before, but given every four hours. During the day many more pieces of membrane were thrown up and the right side of the throat was almost cleared of it, the respiration was better, the hoarseness much less.

On the 16th, I found my patient much better, the pulse not very frequent and the skin pleasant, still some hoarseness and frequent cough. The ammonia mixture to be given occasionally during the day, and between the doses a teaspoonful of syrup of squills. More of the membrane was thrown up, the throat free from it, no operation on the bowels. 17th. Still continued better, no hoarseness, breathing good, bowels confined. Ordered a dose of castor oil, ammonia mixture to be discontinued, syrup of squills to be continued. 18th. The oil has operated three times, the stools are yellow and the patient lively and comfortable, still coughs a little, no more croupy symptoms, ordered squills to be given occasionally, and flax-seed tea. The child continues to improve, having had no return of hoarseness and the cough gradually ceasing.

I think the throwing off of the false membrane in this case may be attributed to the stimulating effect of the ammonia on the mucous membrane, and not to the mechanical efforts of vomiting. Other emetics, as zinc, tartar, and ipecac., failing to produce the effect, though carried even to exhaustion. The ammonia is inhaled at the time the medicine is given, and then again when the patient vomits some of the vapor gets into the trachea, and it is in this way that the ammonia appears to act. Doubtless when the membrane is then loosened by the new action, excited by the hartshorn, the effort of vomiting assists in detaching it from the surface. In many cases of diphthorite, death does not take place until the false membrane extends throughout the trachea and bronchi, and even to the small ramifications of the bronchial tubes. Post mortem examinations always exhibit the smaller bronchial ramifications completely choked with this false membrane. As far as my observations have enabled me to form an opinion, the disease always commences in the fauces and larynx and extends gradually down the windpipe, and it is not until after the patient had been ill for some time, that we find the bronchi and lungs affected. In some cases the patient dies from suffocation before the disease has had time to extend half way down the trachea, and when there is much swelling about the throat, and the false membrane is formed rapidly and thick, the larynx is completely closed by it and the patient suffocates, while the trachea and bronchial tubes are perfectly healthy. It is of great consequence, therfore to apply our remedies whatever they may be, early, for if the patient avoids suffocation in the first stage of the disease, the membrane extends gradually down into the lungs and will soon be beyond our reach.---*Charleston Med. Jour.*

